# Supplementary material for: Transient Activation of Hedgehog Signaling Inhibits Cellular Senescence and Inflammation in Radiated Swine Salivary Glands through Preserving Resident Macrophages
Source: Int J Mol Sci. 2021 Dec 16;22(24):13493. doi: 10.3390/ijms222413493 (PMC8708934; doi:10.3390/ijms222413493)
Supplement: Supplementary file 1 [file ijms-22-13493-s001.zip › ijms-1498822-supplementary Table S1.pdf]

Supplemental Tabel S1. Primers for qRT-PCR.

Genes		Primers
C1q A	F	CACTGACGACGAGAGGCAG
	R	CGTCCCTCCCGTCCAGAT
TNF-α	F	CACCACGCTCTTCTGCCTACTG
	R	GACGGGCTTATCTGAGGTTTGAG
IFN-γ	F	CAAAGATAACCAGGCCATTCAA
	R	TGATGAGTTCACTGATGGCTTTG
IL-4	F	GGCATGTACCAGCAACTTCGTC
	R	AGGTTTCCTTCTCCGTCGTGTT
IL-6	F	TAACCCCACCACAAATGCCG
	R	AGGTGCCCCAGCTACATTATC
P53	F	AAGGGAATTTACGGGCCGAG
	R	CCCCATGCAGGAGCTGTTAC
AIF1	F	AGCCTTCAAACAGAAATACATGGAG
	R	AGCCACTGGATACCTCCTTGAT
ADGRE 1	F	TTACGATGGAGTTCGCCTTGTT
	R	GTGTTATGGTTGCGGATGGTTC
ITGAX	F	CACCTTTGACAGATCCGTGTATTC
	R	TGCTCCTTCCATCATTTCCTTG
HGF	F	TCTCATGCTGCTTCCCCTTC
	R	AGCAAGGCTCAGCCCAATAG
ARG 1	F	TGTCTTCCGTTCAGTAGGTGG
	R	TACACCAGAGTCCTCCAGCC
